# Voxel-wise quantification of myocardial blood flow with cardiovascular magnetic resonance: effect of variations in methodology and validation with positron emission tomography

**DOI:** 10.1186/1532-429X-16-S1-P352

**Published:** 2014-01-16

**Authors:** Christopher A Miller, Josephine H Naish, Mark P Ainslie, Christine Tonge, Deborah Tout, Parthiban Arumugam, Anita Banerji, Robin Egdell, David Clark, Peter J Weale, Christopher D Steadman, Gerry P McCann, Simon G Ray, Geoffrey J Parker, Matthias Schmitt

**Affiliations:** 1 North West Heart Centre, University Hospital of South Manchester, Manchester, UK; 2 Centre for Imaging Sciences & Biomedical Imaging Institute, University of Manchester, Manchester, UK; 3 Institute of Cardiovascular Sciences, University of Manchester, Manchester, UK; 4 Nuclear Medicine Centre, Central Manchester University Hospitals, Manchester, UK; 5 Cardiology Department, East Cheshire NHS Trust, Macclesfield, UK; 6 Alliance Medical Cardiac MRI Unit, University Hospital of South Manchester, Manchester, UK; 7 Siemens Healthcare, Surrey, UK; 8 NIHR Leicester Cardiovascular Biomedical Research Unit and Department of Cardiovascular Sciences, University of Leicester, Leicester, UK

## Background

Quantitative assessment of myocardial blood flow (MBF) from cardiovascular magnetic resonance (CMR) perfusion images appears to offer advantages over qualitative assessment. Currently however, clinical translation is lacking, at least in part due to considerable disparity in quantification methodology. The aim of this study was to evaluate the effect of common methodological differences in CMR voxel-wise measurement of MBF, using position emission tomography (PET) as external validation.

## Methods

Eighteen subjects, including 9 with significant coronary artery disease (CAD) and 9 healthy volunteers prospectively underwent perfusion CMR imaging using a saturation recovery gradient echo sequence acquired at basal, mid and apical left ventricular short-axis levels during adenosine vasodilator stress and at rest, using 0.05 mmol/kg gadolinium-DTPA. Comparison was made between MBF quantified using: 1. Calculated contrast agent concentration curves (to correct for signal saturation) versus raw signal intensity curves; 2. Mid-ventricular versus basal-ventricular short-axis arterial input function (AIF) extraction; 3. Three different deconvolution approaches; Fermi function parameterization, truncated singular value decomposition (TSVD) and first-order Tikhonov regularization with a b-spline representation of the impulse response function. CAD patients also prospectively underwent rubidium-82 positron emission tomography (PET; median interval 7 days) and MBF measurements made using PET and CMR were compared.

## Results

MBF was significantly higher when calculated using signal intensity curves compared to contrast agent concentration curves, and when the AIF was extracted from mid-ventricular compared to basal-ventricular images. MBF did not differ significantly between Fermi and Tikhonov, or between Fermi and TVSD deconvolution methods although there was a small difference between TSVD and Tikhonov (0.0 6 mL/min/g). Agreement between all deconvolution methods was high. MBF derived using each CMR deconvolution method showed a significant linear relationship (p < 0.001) with PET-derived MBF however each method underestimated MBF compared to PET (by 0.19 to 0.35 mL/min/g).

## Conclusions

Variations in more complex methodological factors such as method of deconvolution have no greater effect on estimated MBF than simple factors such as AIF location and observer variability. Standardization of the quantification process will aid comparison between studies and may help CMR MBF quantification enter clinical use.

## Funding

CAM is supported by a Fellowship from the National Institute for Health Research, UK (NIHR-DRF-2010-03-98).

**Figure 1:**
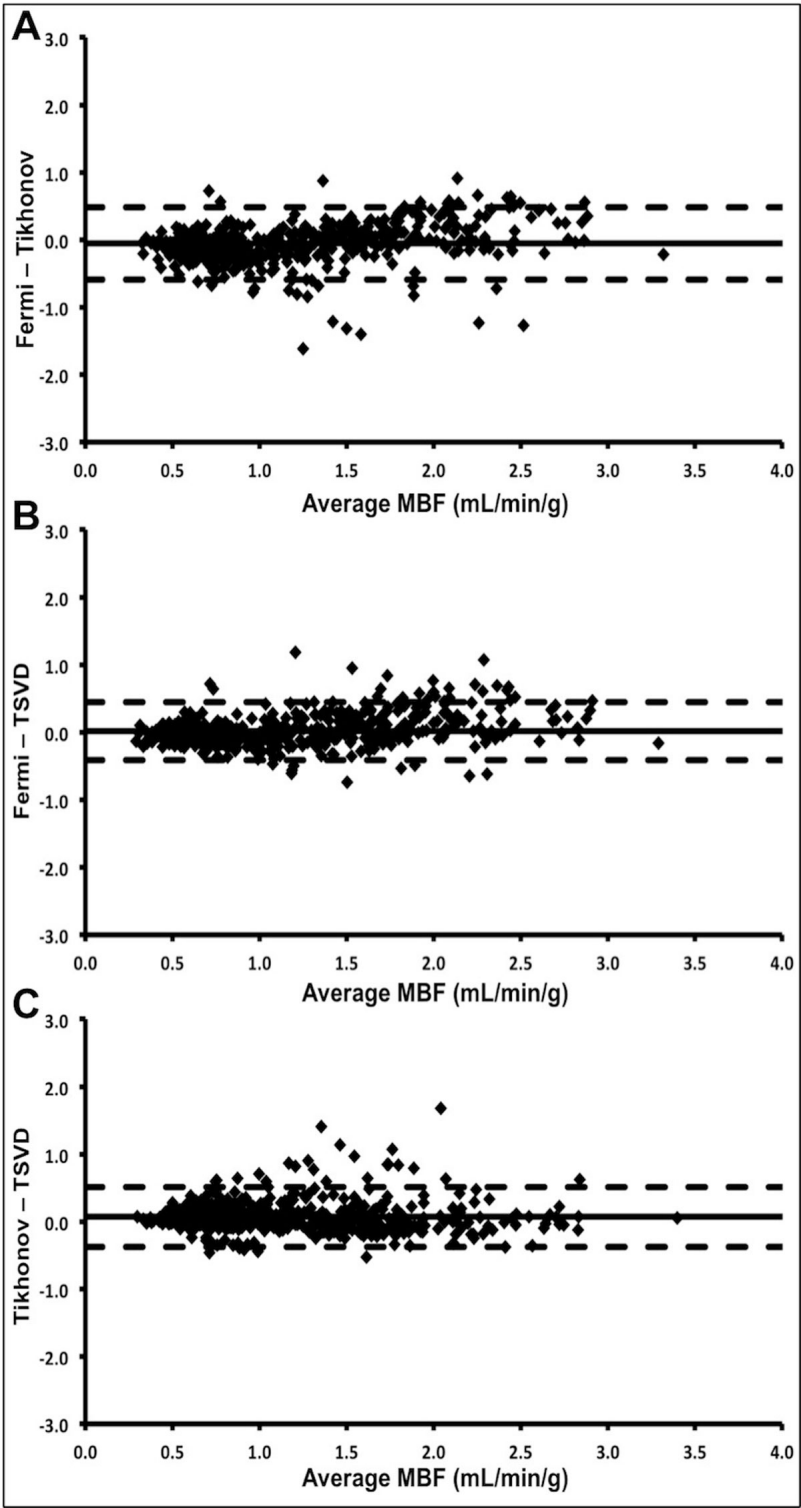
**Comparison of deconvolution methods**. Bland-Altman plots displaying the agreement between myocardial blood flow measured using Fermi function parameterization (Fermi), Tikhonov regularization (Tikhonov) and truncated singular value decomposition (TSVD) methods of deconvolution. Solid line represents mean difference; dashed lines represent ± 2 standard deviations.

**Figure 2:**
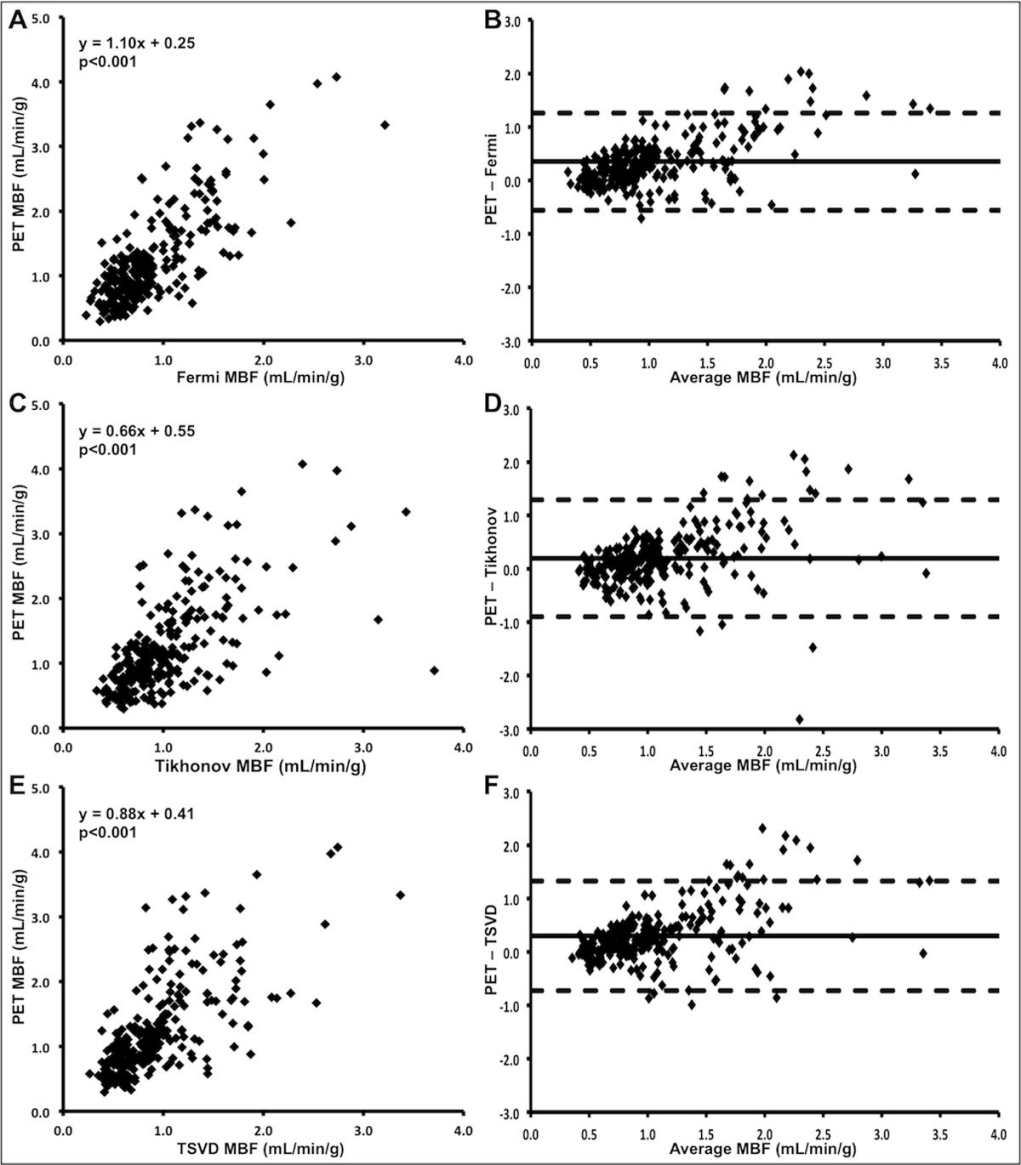
**Comparison of cardiovascular magnetic resonance (CMR) and positron emission tomography (PET)-derived myocardial blood flow (MBF)**. CMR-derived MBF measured using Fermi function parameterization (Fermi, A), Tikhonov regularization (Tikhonov, C) and truncated singular value decomposition (TSVD) (E) deconvolution methods plotted against PET-derived MBF, with corresponding Bland-Altman plots (B, D, F respectively).

